# Strategies for Meaningful Engagement between Community-Based Health Researchers and First Nations Participants

**DOI:** 10.3389/fpubh.2017.00138

**Published:** 2017-06-30

**Authors:** Jaime Cidro, Marion Maar, Sabrina Peressini, Robert J. Schroth, John Broughton, Lisa Jamieson, Herenia P. Lawrence

**Affiliations:** ^1^Department of Anthropology, University of Winnipeg, Winnipeg, MB, Canada; ^2^Human Sciences Division, Northern Ontario School of Medicine, Laurentian University Campus, Sudbury, ON, Canada; ^3^Faculty of Dentistry, University of Toronto, Toronto, ON, Canada; ^4^Faculty of Health Sciences, College of Dentistry, University of Manitoba, Winnipeg, MB, Canada; ^5^Dunedin School of Medicine, Dunedin, New Zealand; ^6^Australian Research Centre for Population Oral Health, The University of Adelaide, Adelaide, SA, Australia

**Keywords:** community research, interpersonal communication, cultural communication, early childhood caries, first nations health research

## Abstract

The Baby Teeth Talk Study (BTT) is a partnership-based research project looking at interventions to prevent early childhood caries (ECC) in First Nations populations in Canada. Community-based researchers (CBRs) conducted preventive and behavioral interventions that targeted expectant mothers and their newborns, over a 3-year period. The work of the CBRs requires a great deal of training and skills to administer the interventions. It also requires a broad set of strategies to meaningfully engage participants to make health-promoting changes in their behavior to prevent ECC in their children. After implementing the intervention, BTT CBRs participated in interviews to explore the strategies they employed to engage participants in the prevention of ECC. CBRs perceived two key strategies as essential for meaningful engagement with BTT participants. First, CBRs indicated that their shared experiences through motherhood, First Nations identity, age, and childhood experience provided a positive foundation for dialog with participants that lead to build trust and rapport. Second, supportive interpersonal and culturally based communication skills of the CBR provided further foundation to engage with participants from a strength-based approach. For example, the CBRs knew how to effectively communicate in ways such as being gentle, non-intrusive, and avoiding any perception of judgment when discussing oral health behavior. In First Nations health research, CBRs can provide an essential link in engaging participants and the community for improvements in health. Researchers should carefully consider characteristics such as shared experience and ability to understand cultural communication styles when hiring CBRs in order to build a solid foundation of trust with research participants.

## Introduction

Community-based participatory research has emerged as a preferred approach to health research with First Nations, Inuit and Métis people (FNIM) (1). The emphasis on involving researchers who are directly from the participating community is a critical component to meaningful engagement with research participants and the community. In First Nations health research, the engagement between participants and researchers has resulted in the common practice of hiring community research assistants (2, 3). The shifts in control in First Nation’s research has resulted in much more participatory, collaborative and in some cases community driven research. The role of the community research assistant has also transformed and their participation begins in the initial design of the research as well as in the analysis of findings. The Baby Teeth Talk project (BTT) (as described below) has embraced this shift in research and employs a Community-Based Researchers (CBRs) approach. In many cases, CBRs have experience in community-based participatory research and often receive further specialized training specific to the project. CBRs are often well equipped to engage in research. In other cases, however, the opportunity to recruit experienced CBRs does not exist, and research projects recruit non-First Nations CBRs.

The Baby Teeth Talk Study is an example of a project employing both First Nations and non-First Nations CBRs. BTT is a tri-country project with Canada, New Zealand, and Australia focusing on four successive interventions to address the high prevalence of early childhood caries (ECC) in Indigenous populations worldwide (4, 5). ECC is characterized as the presence of tooth decay in children aged 5 years or younger (6). The interventions include offering dental care during pregnancy, applying fluoride varnish to the teeth of infants at 6, 12, and 18 months, and using motivational interviewing (MI) and anticipatory guidance to counsel mothers on caring for their children’s teeth (7).

There are two critical elements of community/researcher engagement in this research. The first is the development of partnership-based relationships with the First Nations community partners such as Health Divisions or primary health care providers, which includes adapting the research methods to the sociocultural needs of the community, specifically in the areas of implementation planning at the local level and the dissemination of findings. The second critical engagement component of this research is the enhancing local research skills by hiring and extensively training CBRs to carry out the interventions and collect all the data. To this end, the BTT CBRs attended training sessions held every year in the counseling technique of MI, led by certified experts in MI. This was followed by monthly coaching calls with the MI experts, as well as training sessions on oral health anticipatory guidance, the application of fluoride varnish, and data collection and entry.

In order to enhance and document our methodology, the investigators decided to explore strategies that the BTT CBRs (1) used to establish and maintain relationships with the participants and (2) those that supported effective data collection. This paper describes the experiences of the BTT CBRs in conducting research in the community and their strategies for relationship building with participants during data collection. The focus is not on the BTT participants, but on the experiences of CBRs in the BTT study.

## Background

Health research in First Nation, Inuit and Métis (FNIM) communities has been a challenge both for communities and for the academics conducting research. For communities, a long history of poorly implemented research has resulted in a narrative of negative researcher/community relations with little positive impact or improvement in health conditions for FNIM people (8). However, research in FNIM communities has evolved in recent years. Collaborations between communities and scholars provide many positive examples of the transformation of research in these communities (8). The increasing movement away from curiosity-driven research has resulted in an increasing body of literature and research projects focused on the needs of the community in tandem with the interests of academics. The emergence of Indigenous methodologies are also partially the result of an increasing number of FNIM people completing advanced degrees and contributing important innovations to scholarship. It is also due to the impact of the global Indigenous self-determination movement working at local, national, and international levels. Indigenous methodologies are also being utilized as a more appropriate way of engaging communities in research processes that are reflective of local cultures and traditions of knowledge gathering. This important shift has resulted in a foundational debate on the validity and value of Indigenous methodological frameworks and appropriateness of tools to understand Indigenous cultures and inter-cultural social processes.

Wilson (9) describes the recent phase of Indigenous research taking place that coincided with global Indigenous movements. In Canada, this culminated with the Royal Commission on Aboriginal People (RCAP) report in 1996 and laid the important groundwork for this new phase of research that critically examined the impacts of residential schools, the mass adoption of FNIM children out of their communities in the 1960s, and the negation of treaties through natural resource extractive activities. Social activists of both FNIM and non-FNIM people embarked upon this type of research resulting in the creation of an extensive set of recommendations that sought to redress the unequal structural relations of FNIM people to the Canadian government and society. The First Nations Regional Longitudinal Health Survey was conducted in 1997 in response to the recognition that health and well-being information was lacking from major national health surveys and that First Nations and Inuit need to control their own data (10). This was designed and delivered for First Nations, by First Nations people (the Inuit withdrew their participation and developed their own survey tool). Indigenous scholars worldwide began to emerge strongly during this period and with it, the introduction of Indigenous methodologies, most notably in the work of Maori scholar Linda Tuhiwai Smith (11).

### Shifts in Research Control

Indigenous paradigms in the academy took a further shift with the release of *Decolonizing Methodologies: Research and Indigenous Peoples* (11) where western methods of inquiry were challenged as perpetuating ongoing colonization of Indigenous peoples across the globe. Smith (11) questions the assumption that research and research methods are “culture free” or “value free” and that researchers occupy a moral high ground of objectivity. This important contribution to legitimizing indigenous research methodologies has influenced the current stage of indigenous research. Presently, Indigenous scholars are openly acknowledging and illuminating their Indigenous worldview using their own data collection methods and research paradigms (9). Brant Castellano’s (12) article on the research ethics published in the inaugural edition of the Journal of Aboriginal Health, paved an important path to exploring the rigor placed on scholars who wish to research in FNIM communities. First Nations scholars, like Castellano are now using the academy as a way to explore their own cultural pedagogy and challenge historical assumptions. Scholars today are taking direction from FNIM communities on research topics that are pertinent and contribute to self-determination. This is a marked shift from the earlier phases of research on FNIM people, which sought to control and assimilate individuals and whole communities into the mainstream.

In Canada, the movement toward more appropriate research ethics practices has also shaped Indigenous health research. At the government funding level, the Tri Council Policy Statement: Ethical Conduct for Research Involving Humans, Chapter 9: Research Involving First Nations, Inuit and Métis People of Canada (1) has provided an important framework for conducting respectful research with FNIM communities and individuals. In health research, the Institute of Aboriginal People’s Health, which is one of the 13 institutes of the Canadian Institute of Health Research, focusses on meaningful research that addresses the health needs of the community. On a community level, many First Nations communities have established their own research offices that provide a variety of functions including research ethics specific to the community, community approval, and assistance with research. For example, Six Nations of the Grand River in Ontario has a research ethics committee with their own set of research ethics principles guiding how researchers and the community interact in projects (13). First Nations communities in Manitoulin Island (Ontario) have established a similar set of principles and research review committee called the Manitoulin Anishinabek Research Review Committee (14). In Manitoba, the Chiefs in Assembly in 2007 formally committed to self-determination in respectful research relationships by embedding three principles: (i) free, prior, informed consent (on individual and collective levels); (ii) First Nations Ownership, Control, Access and Possession (OCAP™) principles of ownership, control, access and possession of their own data, and (iii) First Nations ethical standards (15).

### Research Skill Enhancing or Building in the Community

One important aspect of the OCAP™ principles includes enhancing or building research skills in the community. The nature of academic research has followed the typical trajectory of an academic lead investigator, with graduate students and in some cases undergraduate students collecting and analyzing data, and in other cases, the recruitment of local key informants or community members to assist with the research. However, in FNIM communities, the role of community members as ancillary to the research process has diminished and instead taken center stage. As the OCAP™ principles reinforce, the power relationship between the academic community and the FNIM community has now shifted and the responsibility of the researcher in providing services such as training, employment and community skill building in research are now an increasing requirement for communities to even consider participation in research. As Jacklin and Kinoshameg (16) describe the process of including community members as members of the research team is critical in the development of research tools, as well as the actual data collection because it makes “participants feel confident in the promise of complete confidentiality” (16) (p. 57). In reference to Australian Aboriginal researchers, Laycock (17) states that community capacity building to do health research resulting in practical and positive change requires “being serious about building quality training and offering real support for Indigenous researchers” (17) (p. 9).

### Insider Research

Laycock (17) also describes some of the complexities associated with insider research. In many cases, researchers may anticipate that Indigenous researchers are ideally situated to “open doors” in Indigenous communities; however, this is a simplistic and an unreasonable assumption that does not take into account extra pressure on inexperienced researchers. Issues such as family background, kinship links, gender, political connections are another layer of expectations that Australian Aboriginal researchers are burdened with that non-Indigenous researchers do not experience (18).

The role of mutual ethnicity as a point of connection for researchers is a central component of the native insider approach because it also provides a source of “crucial responsibility…in doing this type of research” (19) (p. 220). When there is no shared identity between the researcher and the respondents, other points of connection still coincide with the native insider approach. As Goldade (20) describes having a newborn baby brought along in the interviews made recruiting participants less challenging, and helped “knock down trust barriers, thus smoothing the work of eliciting narratives on sensitive, yet pertinent topics confronting my informants around reproduction, reproductive health, and motherhood” (20) (p. 53).

Community researchers in rural and remote communities are also faced with different challenges than those in larger urban centers. Fears from participants about lack of confidentiality from “insiders” or CBRs from the FNIM community are common in many small communities but are also symptomatic of larger issues of lateral violence, which plague many communities. The effect of lateral violence is expressed in various ways such as gossip, perpetual social infighting, suspicion, and mistrust of others (21). This is compounded in reserve communities where people are further physically and socially isolated and certainly can impact the success of research projects.

## Methodology

In the BTT Study undertaken in Manitoba and Ontario, each individual community engaged in a process of relationship and trust building through pre-existing relationships with the principal investigator of the BTT study (HPL) and co-investigators (22). The hiring of community researchers or CBRs, who in most cases were First Nations people from the communities was also critical in promoting the research project. Due to the nature of the research, significant training with the CBRs has been an ongoing process to ensure that the intervention methods being utilized were consistent between research sites as well as building the necessary research skills for CBRs.

A key component of this research project has been the CBRs. In each community, a CBR was hired to deliver the interventions and collect the data. In most cases, the CBRs are from the communities and are First Nations (six out of eight of the CBRs). In cases where the CBRs are non-First Nations (two of the eight CBRs), they have a significant connection to the community or have an understanding of the larger social and cultural determinants of health where First Nations are concerned. In all cases, CBRs were initially provided with training in the research methodology and were physically gathered together for ongoing training every year for 3 years. In addition, training continued through the use of conference calls with a trainer, as well as updates with the Principal Investigator.

The BTT project takes a participatory approach in recognizing how the relationship between the investigators, the CBRs, and the BTT participants are realigned. BTT CBRs come to the project with their own theoretical and experiential knowledge, which has helped to shape the success of retaining our BTT participants by engaging in authentic relationships. The research also hinges on the development of authentic research relationships (23), which requires meaningful and collaborative relationships so the investigators can learn enough about the community to interpret and analyze the data respectfully and appropriately. A key element of this is our CBR who are the lens in which we understand the experiences of BTT participants. The critical nature of the CBRs in this project renders it important to explore their experiences in the project to understand the strategies they use to engage with participants. A separate research project was subsequently undertaken to explore these strategies and approaches. Research ethics approval was obtained through the home institution of the interviewer and co-investigator from the University of Winnipeg Research Ethics Board for this particular research.

The participants were informed by email by the BTT Principal Investigator of the request to be interviewed and were informed that their participation was not mandatory and there would be no expectation to participate. Despite this, all eight CBRs at the time contacted the BTT PI and volunteered to participate. An experienced Indigenous qualitative researcher and coinvestigator on the BTT project conducted semi-structured individual interviews with all of the eight of the CBRs. A semi-structured or conversational approach was used because of the open-endedness and ability for the interviewer to delve into topics that may not have been anticipated and bring out how the interviewees themselves understand and interpret issues and event (24). This approach is important in conducting these interviews because it provides the necessary environment for the participant to have a large amount of control over what they choose to discuss and the emphasis they would like to place on these particular topics.

Interviews were conducted either on the phone or in person. Interviews were typically an hour in length and were audio recorded, and transcribed. All of the participants were familiar with the interviewer/coinvestigator through the training sessions. Consent was obtained prior to the commencement of the interview. When the interviews were not face-to-face, the consent form was read out loud and participants indicated their agreement to participate and it was noted in written format that oral consent was received. Written consent was obtained for the two interviews that were conducted face-to-face. The interview questions focused on issues of trust with participants and the role of being a First Nations or non-First Nations researcher in the area of FNIM health. Specifically, topics explored included: the importance of working in the area of FNIM health for the CBRs, challenges, and opportunities of being a First Nations person and working in the community, trust as an issue for both researchers and participants, and the importance of having a dental health background in this research.

The interviews were transcribed verbatim and returned to all of the participants to ensure that information was correct. Utilizing the principles of grounded theory, an initial coding framework was developed by the lead author and interviewer (Jaime Cidro) immediately after conducting the interviews. This initial coding, a focus on actions rather than themes and topic was developed to avoid the tendency of making “conceptual leaps” and “adopt extant theories” prior to the analysis (25) (p. 117). Three qualitative researchers then tested the initial coding framework independently by reviewing all the transcripts. The researchers then evaluated the fit and usefulness of the codes (26) and adjusted the framework accordingly. The research team independently coded all the transcripts using a selective approach to identify codes that appeared frequently and seemed most revealing as it pertained to the original research question (27). The research team reviewed all independently coded transcripts together and any outstanding inconsistencies were identified and discussed. The research team engaged in constant comparison between the categories as well as with the other authors, which entailed “sensitivity to differences between emerging concepts/categories” (26) (p. 515). Agreement was reached at all points where inconsistencies were noted.

A draft copy of this paper was sent to the CBRs as a way to ensure trustworthiness. Participants were asked to review the draft paper to ensure that the authors interpreted the social reality of the CBRs in their interviews accurately. Member validation is an important part of confirming the account is consistent with the ways the participants see the world (27).

## Limitations

The BTT CBRs are used to working one on one with expectant mothers and her children in a face-to-face setting. They develop relationships by sharing considerable periods of time talking about in-depth personal issues relating to oral health and infant development. However, when interviewing the BTT CBRs, the majority of interviews were conducted over the phone, which may have influenced the ability of the CBRs to provide thorough responses. The CBRs were provided with a copy of the interview questions in advance to allow for time to reflect on their responses. Out of the eight CBRs interviewed, two were not First Nations. Early discussions were held to determine whether excluding the non-First Nations CBRs would provide us with a clearer explanation of the experiences of CBRs; however, it was determined that the two non-First Nations CBRs had considerable experience working in a First Nations/FNIM community, and while not “members” still had some important experiences to share.

## Results and Discussion

All eight of the CBRs revealed an intense dedication to working with participants in their communities. Two themes that emerged with all the CBRs included the use of shared experience as a foundation to develop trust and establish rapport, and the use of cross cultural communication to build relationships. Both of these themes are discussed using the voices of the CBRs directly.

### Shared Experience

The Baby Teeth Talk Study participants were all women and started out in the research project while they were pregnant, which provided an important foundation for the development of shared experiences with CBRs. Meaningful engagement with participants was most prominently established by having commonalities. For example, all of the CBRs were women and most of them have children, including newborns and infants, which provided a good foundation for connecting with the female participants. In most cases, the CBRs are in the same age range (25–35 years old) as the participants. In the cases where participants and CBRs are from the same community, they knew each other from similar social settings as well as through family networks. For the six CBRs who were First Nations, they shared a common First Nations identity with their participants, which also resulted in shared communication styles and intrinsic understanding of community life.

One issue that was discussed in this research was First Nations identity. It was considered as an important foundation for establishing relationships. The CBRs noted that their interactions with their research participants were guided by her traditional teachings: “I deal with them with respect and dignity. I use the seven grandfather teachings; honesty, humility, truth, wisdom, love, respect, and bravery.” This theme continued through the second phase of the research. Having a cultural awareness was shown to be an important tool for successful community research because an established understanding of challenges in the community also provided a good foundation for trust building. As one respondent indicated: “I’m a First Nations person, I’m from the area. They know me, they know my background… I believe that helps with the trust.”

The majority of the First Nations CBRs had similar childhood experiences and also had young children. CBRs were able to use this as a tool to develop a relationship with the participants. This approach is described by one CBR: “I share my story and use that as a way to build trust.” Another respondent indicated the same idea: “We come from the same place, we have experienced the same issues, and we share the same culture, so it makes it easier.” During the research, some of the CBRs were pregnant and gave birth. While this is consistent with the “insider” approach described by Ogawa (19), it is a unique type of researcher positioning described by Goldade’s (20) experience as a new mother talking to other mothers about issues relating to infant health. The role of being a mother, especially a first time or new mother provided an important way for the CBRs to connect with participants. Several CBRs discuss how this shaped their connections with participants:

I was pregnant at the time I was recruiting as well, so I felt I had more of a connection with the women. I thought I was more relatable with me being pregnant. And later on after I had my baby…I brought the baby with me. I had hoped that it would make a difference bringing the baby along.

When I was pregnant, and I was speaking to women in the interviews, I was using myself as an example, saying that I didn’t know a lot of the stuff until I started with the study. To show I guess that I…. I don’t know how to explain it. To show I guess that it’s ok that you didn’t know it. It doesn’t mean that you are ignorant or stupid and that it is actually more common not to know the stuff in the community.

In many communities, participants are initially reluctant to talk to CBRs. Even though participants have agreed to participate in the project, there are often other “gatekeepers” that the CBRs must go through in order to engage with the participant. One CBR shared an interesting story regarding connecting with the grandmother of a participant:

There was one time I knocked on the door and there was a grandmother that answered the door. She didn’t really want to let me in. She kind of opened the door and kind of looked at me and asked me who I was and what I was doing. After a while I introduced myself, she invited me in. Then we started talking about my sister, she knew my sister. I told her I was from (First Nation) and after that she was more welcoming. We talked about other stuff, and she asked about my sister and the visit was so awesome. After a while, when I told her who I am, where I’m from, and she asked me if I knew this person, we just had such a good visit!

The complexities of being a “native insider” as Laycock (17) describes is often nuanced and requires the researcher to develop ways to engage in multiple ways. CBRs often connected with participants by sharing personal information with them to “level the playing field.” This is an important part of ensuring confidentiality (16). By giving up personal information to participants and making themselves vulnerable, it provides some assurances beyond the consent form of confidentiality. This was described by one CBR: “I share my story and use that as a way to build trust. I also make sure I never tell anyone else what the participants tell me.” The need for FNIM researchers to make themselves vulnerable by sharing personal stories about themselves is certainly one of the challenges that Indigenous researchers face (17), and it is important for those researchers to understand that this may be something they face in communities in which they are working.

One of the CBRs discussed sharing a childhood memory with her participants, which was a powerful way of connecting and shows the culturally specific context and understanding that is required for this project:

One story I find myself sharing all the time is on the experience I had as a child receiving dental treatment that was really negative. I find the women also have this experience. They also had a lot of general anesthesia, and their first experience with the dentist was usually surgery and extracting teeth. So we didn’t have good oral health experiences. Once we started talking about these experiences, they begin to realize how it has affected them now in terms of not going to the dentist’s office.

### Cross Cultural and Interpersonal Communication

Shared culture facilitates having shared experiences; this was less of a challenge for the First Nations CBRs than the non-First Nations CBRs for obvious reasons. Shared experiences such as motherhood and First Nations identity were not shared by the two non-First Nations CBRs, both of these CBRs were living in the large urban centers (Winnipeg and Toronto) and, instead, relied on other mechanisms to establish a relationship with their participants. Having an understanding of how different people communicate based on cultural differences and historical experiences and understanding how to adapt interpersonal communication approaches to develop rapport and trust was an issue that the non-First Nations CBRs undertook as a strategy.

The CBRs discussed the importance of making the participants feel comfortable, which in part is to ensure that they are not feeling judged. BTT participants can often feel judged about their skills as a parent, especially given the long history of child and family services intervening in many FNIM communities. CBRs must be cognizant of ensuring that participants are comfortable. One respondent described her approach as “learning to be open and relating to them. It’s important to take the lab coat off. I like to treat it as a meeting of two girls chatting over coffee.” Another respondent described this similarly:

It’s important to not be judgmental, and just to be willing to share your own experiences. I think you really have to be gentle and open with native women, Native people. Not to be authoritative, not to sound like you are preaching or like you have to teach them, but just that you are there for them.

To be effective community researchers, it is important to hire and/or train CBRs to consider the larger historical context of colonialism when engaging with participants. The need for culturally based communication styles is even more pronounced in remote communities where community members are less likely to engage with people who are not from their communities on a regular basis. One respondent from a remote community described the issues of communication:

Around these areas, native people find non-native people or white, find them intimidating. It is almost kind of like authoritative figures. Not pushy, but like that same residential school mentally I guess. They have almost a fear, or they don’t think of themselves on the same level maybe.

This type of sentiment is at the heart of why CBRs, even if they are non-First Nations or from the communities, requiring a deep sense of awareness of the larger context of the communities in which they are working. This was not a formal part of our vetting process in hiring CBRs; however, all of our CBRs had an understanding of this context, which was either inherent or learned through previous experience.

The literature supporting FNIM control of research highlights factors such as working in a participatory manner with community and shifts in control in which there is a lack of literature that describes the features that researchers should be considering when hiring local researchers who are the ones carrying out the majority of data collection. While there was no surprise that shared experiences, being able to identify with participants and build relationships was a key part of the success of the research, there are gaps in the literature to explain these features as a key determinant and outcome of successful research relationships in FNIM communities. Considering the large scope of the BTT project, it was considered worthwhile exploring this topic rather than “taking it for granted” that these factors were important.

## Conclusion

The role of CBRs has become increasingly critical for the successful engagement with FNIM communities, who have long suffered from a history of poorly implemented research done by “outside” researchers. BTT has taken a community-based research approach, which is centered on the development of culturally appropriate research tools and dissemination, and engagement with participants in ways that are meaningful through the use of CBRs. The CBRs in BTT have recruited more than 500 participants over a period of approximately 14 months and the interventions are nearly completed. The effectiveness of the CBRs can be attributed to several factors. The importance of having a shared experience between the CBRs and the research participants paved the way for successful engagement. Shared identity and childhood experiences and relatability through motherhood were all important shared experiences that provided CBRs with a foundation to build trust and understanding. This foundation was further enhanced through cross cultural and interpersonal communication styles. BTT CBRs know how to effectively communicate with participants given their experiences with non-FNIM people, the larger history of colonial interactions, and the cultural modes of communication.

Given the long history of negative relations between researchers and FNIM communities, understanding the role of CBRs as being critical to engagement with community and participants cannot be understated. Whether CBRs are FNIM and from the community where research is taking place, or non-FNIM, all CBRs need to understand that research is not solely about the collection of data. Meaningful engagement requires CBRs to draw on their own foundation as people, using their own sensibilities, sensitivities, and experiences to develop and build relationships with participants. It is important not only for the research task at hand but also for the larger trajectory of research in FNIM communities and the ongoing development of healthy, collaborative relationships with researchers. Future research could consider whether health research in FNIM communities or other populations should solely rely on CBR from those populations specifically, or are there other more salient factors that need to be considered such as personal sensibilities and sensitivities and ability to communicate in different cultural settings.

## Ethics Statement

Research ethics approval was obtained through the home institution of the interviewer and coinvestigator from the University of Winnipeg for this particular research. The participants were informed by email by the BTT Principal Investigator of the request to be interviewed and were informed that their participations were not mandatory and there would be no expectation to participate. Consent was obtained prior to the commencement of the interview. When the interviews were not face-to-face, the consent form was read out loud and participants indicated their agreement to participate. Written consent was obtained for the two interviews that were conducted face-to-face.

## Author Contributions

JC led this part of the BTT Study, initiated the analysis, and conducted the majority of the writing. MM, SP and HPL independently coded and participated in data analysis and interpretation of the results. HPL is the BTT Study PI in Canada and RS is a co-I on the BTT Study (Canada). They were all involved in revising the manuscript. LJ and JB are the BTT Study PIs in Australia and New Zealand, respectively.

## Conflict of Interest Statement

There were no financial, commercial, or other relationships that might be perceived by the academic community as representing a potential conflict of interest represented in this work. The authors, or authors’ institutions did not receive payment or services from a third party for any aspect of the submitted work. There are no financial relationships with entities that could be perceived to influence, or that give the appearance of potentially influencing in this paper. There are no patents and copyrights, whether pending, issued, licensed and/or receiving royalties relevant to this work. There are no other relationships or activities that could be perceived as having influenced, or that give the appearance of potentially influencing in this paper. The reviewer, DC, and handling editor declared their shared affiliation, and the handling editor states that the process nevertheless met the standards of a fair and objective review.
